# Mast Cells and Influenza A Virus: Association with Allergic Responses and Beyond

**DOI:** 10.3389/fimmu.2015.00238

**Published:** 2015-05-18

**Authors:** Amy C. Graham, Rachel M. Temple, Joshua J. Obar

**Affiliations:** ^1^Department of Microbiology and Immunology, Montana State University, Bozeman, MT, USA

**Keywords:** mast cell, mast cell activation, influenza A virus, dengue virus, inflammation, degranulation, viral infection, viral immunology

## Abstract

Influenza A virus (IAV) is a widespread infectious agent commonly found in mammalian and avian species. In humans, IAV is a respiratory pathogen that causes seasonal infections associated with significant morbidity in young and elderly populations, and has a large economic impact. Moreover, IAV has the potential to cause both zoonotic spillover infection and global pandemics, which have significantly greater morbidity and mortality across all ages. The pathology associated with these pandemic and spillover infections appear to be the result of an excessive inflammatory response leading to severe lung damage, which likely predisposes the lungs for secondary bacterial infections. The lung is protected from pathogens by alveolar epithelial cells, endothelial cells, tissue resident alveolar macrophages, dendritic cells, and mast cells. The importance of mast cells during bacterial and parasitic infections has been extensively studied; yet, the role of these hematopoietic cells during viral infections is only beginning to emerge. Recently, it has been shown that mast cells can be directly activated in response to IAV, releasing mediators such histamine, proteases, leukotrienes, inflammatory cytokines, and antiviral chemokines, which participate in the excessive inflammatory and pathological response observed during IAV infections. In this review, we will examine the relationship between mast cells and IAV, and discuss the role of mast cells as a potential drug target during highly pathological IAV infections. Finally, we proposed an emerging role for mast cells in other viral infections associated with significant host pathology.

## Introduction

Influenza A virus (IAV) is a common human respiratory pathogen, which causes annual seasonal infections with a low frequency of morbidity and mortality, usually limited to the young (<5 years) and the elderly (>65 years) populations. Importantly, IAV has the potential to cause global pandemics, which can significantly increase morbidity and mortality throughout the entire population (1). In the past century, there have been four major IAV pandemics: the 1918 H1N1 “Spanish” influenza, the H2N2 “Asian” influenza in 1957, the H3N2 “Hong Kong” influenza in 1968, and more recently, the reemergence of a pandemic H1N1 (H1N1pdm) influenza in 2009 (2). Moreover, significant spillover infections from the zoonotic avian reservoir of IAV continue to have an impact on the human population, including the current avian H5N1 and H7N9 IAV outbreaks in Southeast Asia (3). To date, these H5N1 and H7N9 outbreaks have remained a spillover event, but the potential of these novel avian IAV strains to develop the ability to efficiently transmit human-to-human through aerosol droplets exists (3–5); thus, increasing the threat of new global pandemics.

As an RNA virus that lacks proofreading capabilities, IAV has a high mutation rate, resulting in significant antigenic drift in the immunodominant hemagglutinin (HA) and neuraminidase (NA) proteins. Furthermore, owing to its segmented genome, IAV can undergo genetic reassortment (antigenic shifts), resulting in novel IAV strains with the potential to rapidly transmit between humans to cause a new pandemic. Given these factors, the next pandemic IAV strain is nearly impossible to predict, leading to many challenges in vaccine development. Current vaccine strategies take approximately 6 months for production. During the 2009 H1N1 pandemic, this delay resulted in no effective vaccine being available for the first wave of the pandemic (2). Thus, it is necessary to find alternative ways to alleviate and treat IAV-induced disease during the early wave(s) of a novel pandemic IAV outbreak.

Antiviral drugs are an obvious front line of defense against the emergence of novel IAV strains. Currently, two main classes of antiviral drugs are approved to treat IAV-infected patients. The first class of antiviral drugs targets the M2 ion channel (amantadanes), which is important for virus uncoating. However, amantadanes are no longer recommended for prophylaxis or treatment of IAV due to widespread resistance among current human seasonal H1N1 and H3N2 isolates (6–8). The second class of antiviral drugs targets the enzymatic active site of the viral NA. The viral NA is a sialidase capable of hydrolyzing terminal sialic acid residues from glycoproteins and glycolipids. The NA is crucial in allowing the IAV to traverse the glycan rich soluble mucins in the respiratory tract, as well as allowing newly formed virions to be released from host cells, to be shed into the extracellular space for dissemination within a host and transmission between hosts. NA inhibitors are becoming of limited efficacy as well, due to emerging resistance among IAV isolates found in humans and the requirement for early administration (within 48 h of the presentation of symptoms) for maximal effectiveness (2, 7, 9–12). Therefore, additional antiviral drugs are required to limit IAV-induced disease and fight the spread of IAV. Numerous drugs are currently in development, which target viral entry, viral transcription, or host factors necessary for IAV replication (9). However, the effectiveness of these drugs against IAV in the clinical setting is unknown.

An alternative front line defense against the emergence of novel IAV strains is to target the inflammatory pathways that lead to lung damage and loss of function (13, 14). Alveolar epithelial cells, endothelial cells, tissue-resident alveolar macrophages, dendritic cells, and mast cells protect the lungs, as these cells are readily able to respond to invading pathogens. Pandemic strains of IAV, including the 1918 “Spanish” influenza and the 2009 H1N1pdm influenza, and spillover infections with avian IAV isolates can produce excessive tissue damage and pathological changes to the lung architecture (1, 15, 16). Current evidence suggests the lung injury induced during IAV infection is the result of excessive leukocyte infiltration and an exaggerated inflammatory cytokine response that is disproportionately high relative to the level of viral replication, which has been termed a “cytokine storm” (16–21). Selectively dampening the inflammatory response in mice has been shown to increase survival following IAV infection without impairing viral clearance (16, 17, 19–22). Thus, understanding the inflammatory cascade responsible for the immunopathology observed following IAV infection is imperative for the development of novel immunotherapeutics aimed at limiting IAV-induced disease and pathology.

Macrophages and neutrophils are recruited at excessive levels following infection with the 1918 or H5N1 influenza strains (16). More recently, it has been demonstrated that mast cells play a pivotal role in initiating and/or amplifying the immunopathological “cytokine storm” and inflammatory leukocyte recruitment in the respiratory tract during IAV infection (23–25). Mice infected with either H1N1 or H5N1 IAV demonstrated elevated levels of inflammatory cytokines and chemokines during infection. Conversely, mice lacking mast cells or treated with mast cell stabilizing agents show a reduction in the levels of these inflammatory mediators that correlates with a decrease in the recruitment of inflammatory cells to the lungs during infection (23, 24). Therefore, it is crucial that the individual and collective roles of these inflammatory cells, with each other and with the epithelial and endothelial compartments, during pathological IAV and other pathological viral infection, be more thoroughly examined.

### Mast cell biology

Mast cells are tissue resident, granule-containing cells capable of regulating both the innate and adaptive immune response (26). Enrichment of mast cells at environmental interfaces allows these cells to be among the first to respond during pathogen invasion, along with dendritic cells and epithelial cells (27). Moreover, mast cells are typically situated near blood vessels, lymphatics, and nerve endings, enabling them to have long range effects on the host response to pathogens (27, 28). As such, mast cells are critical to immune surveillance, eliciting an immediate reaction to invading pathogens and initiating an appropriate innate and adaptive immune response.

#### Phases of the Mast Cell Response

**Figure 1:**
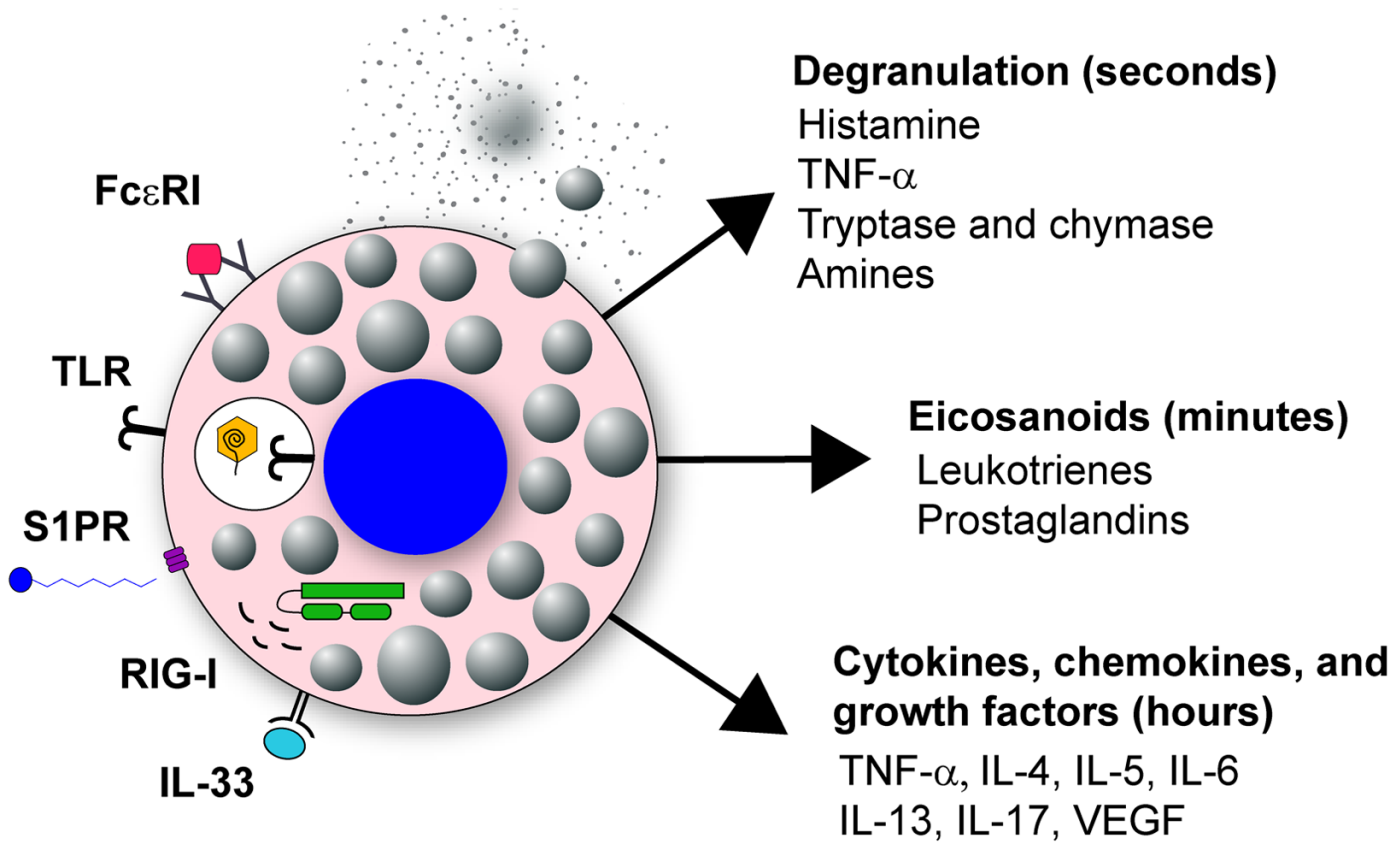
**Mast cell activation in response to viral infection**. Mast cells are classically known for their response to polyvalent cross-linking of IgE in the FcϵR1 receptor, which is important in protective immunity to helminth worm infection and pathologically associated with allergic disease. However, mast cells also are important tissue sentinel cells for initiating inflammatory response to pathogens. Mast cells can recognize and respond to viruses through several different receptors. These receptors include TLR signaling, such as TLR3 detection of dsRNA, sphingosin-1-phosphate (S1P) binding to its receptor S1PR, and RIG-I recognition of uncapped vRNA. Engagement of these receptors results in mast cell activation leading to immediate degranulation, the *de novo* synthesis of eicosanoids within minutes of activation, and the *de novo* synthesis of numerous cytokines, chemokines, and growth factors within hours of activation.

**Figure 2:**
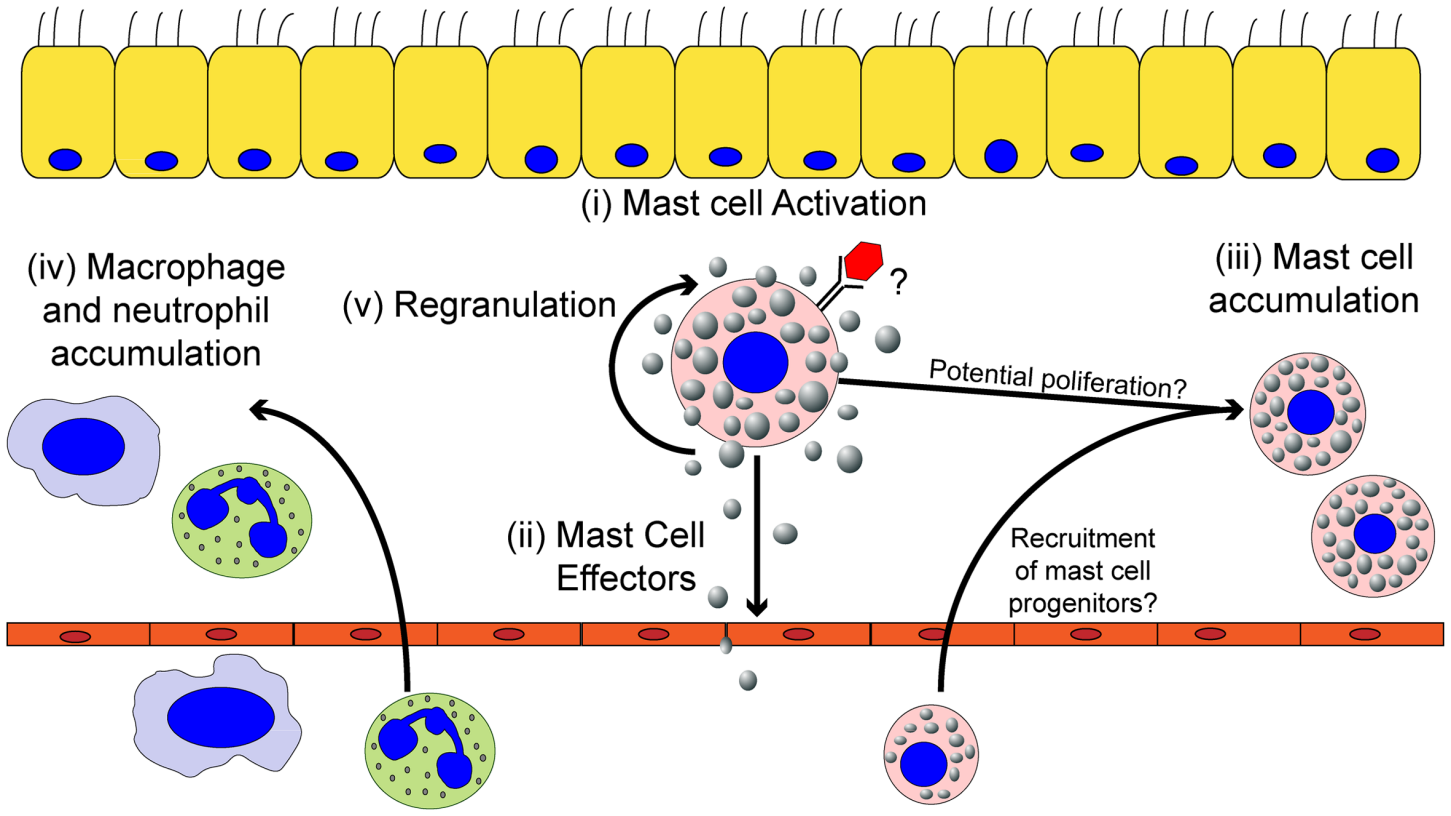
**The effects of mast cell activation on the inflammatory environment induced by viruses**. Within the tissues, mast cells can be activated by viruses (i) resulting in the secretion of effector molecules (ii). Mast cell-derived effector molecules act within the local tissue environment or at distal site to mediate the accumulation of mast cell progenitors (iii) and leukocytes (iv) to the site of infection. Mast cell accumulation in the infected tissues could be due to either the recruitment and differentiation of mast cell progenitors to the infected tissue and/or proliferation of the tissue-resident mast cell population. Mast cell activation can participate in limiting viral replication in the local tissue and viral dissemination, but if left unchecked can cause significant tissue damage, vascular leakage, and tissue edema. Finally, activated mast cells can survive the pathogenic insult and replenish mast cell granules to return the mast cell to a basal state to survey the tissue for future pathogenic insults (v).

Mast cells have two distinct phases of activation: immediate degranulation, resulting in the release of pre-synthesized mediators, and delayed secretion of secondary *de novo* synthesized mediators (27, 29, 30). The delayed secretion of secondary *de novo* effector molecules produced by mast cells can be further segregated into two classes: (1) prostaglandins and eicosanoids released within minutes of activation, and (2) cytokines, chemokines, and growth factors that are released within hours of stimulation (Figure 1). Together, these mast cell outputs can increase epithelial and endothelial cell permeability and activation state, which together with chemotactic molecules, result in increased inflammatory cell recruitment to infected tissues (Figure 2).

Mast cell granules contain histamine, TNF-α, amines, β-hexosaminidase, serotonin, antimicrobial peptides, and proteases (tryptases and chymases) bound to either heparin or chondroitin sulfate through electrostatic interactions (29, 31–33). Upon stimulation, the granules are released from the cell via a calcium-dependent exocytosis process. Once expelled, the granules can either discharge the stored mediators into the immediate environment or intact granules can travel through the bloodstream and lymphatics, acting as a signaling mechanism to activate and recruit other cells to the infected tissue (34, 35). Histamine is a potent inflammatory molecule, which increases vascular permeability, induces vasodilation, and stimulates bronchial smooth muscle contraction. The inflammatory cytokine TNF-α promotes local and systemic inflammation while enhancing neutrophil recruitment to the site of infection. Granule proteases are capable of increasing vascular permeability and enhancing the recruitment of neutrophils to the site of inflammation (36–39), or can act directly to detoxify toxic proteins (40–43). Interestingly, the local homeostatic cytokine milieu of a tissue modulates the precise granule components, allowing mast cells to adapt to their local environment to mount a tissue appropriate inflammatory response (44, 45). Following activation, mast cells are unique in that they replenish their granules, usually within weeks of activation (46, 47). This ability to regranulate allows mast cells to tailor the composition of their granules, and thus be more prepared for reinfection (Figure 2) (27).

After the immediate mast cell degranulation response, the arachidonic acid-dependent inflammatory mediators, such as leukotrienes and prostaglandins, are rapidly produced and released from mast cells due to enzymatic, rather than transcriptional, changes within the mast cell (48). These lipid mediators contribute to local vascular permeability, tissue edema, and the recruitment of neutrophils and other inflammatory cells (49–51).

Finally, *de novo* synthesized cytokines, chemokines, and growth factors are released, hours following activation through transcriptional and translational up-regulation. The multitude of cytokines, chemokines, and growth factors released by mast cells include *de novo* synthesized TNF-α, IL-4, IL-5, IL-6, IL-13, IL-17, and VEGF (32, 52). These mediators activate tissue-resident cells, while recruiting additional effector leukocytes and lymphocytes to maintain the inflammatory state for a prolonged time. In conclusion, through the release of numerous chemotactic factors and vasodilators, mast cells are optimized for the rapid initiation and propagation of an acute inflammatory response through degranulation, production of bioactive lipids, and secretion of cytokines and chemokines. The resulting leukocyte and lymphocyte infiltrate can then help to maintain the inflammatory state if the infection persists (Figure 2).

#### *De Novo* Mast Cell Recruitment during Inflammation

In addition to tissue-resident mast cells, mast cell progenitors can be recruited to sites of acute or chronic inflammation. How the recruitment of these mast cell progenitors is regulated is just now beginning to be understood. Mucosal mast cells (MMC), the dominant type of mast cell in the lung, develop from the bone marrow as mast cell progenitors (53). In an asthma model, following aerosolized challenge with ovalbumin, mast cell progenitors are rapidly recruited into the lungs, peaking day 1 after challenge (54). In this ovalbumin-induced allergic airway inflammatory disease, multiple pathways are critical for mast cell progenitor accumulation in the lungs. Integrins α_4_β_1_ and α_4_β_7_ regulate the migration of mast cell progenitors to the lungs through VCAM-1 interactions (55). Moreover, CXCR2 expression in a radio-resistant cell population is important in regulating mast cell progenitor recruitment to the lungs, likely through its regulation of VCAM-1 on the pulmonary endothelium (56). NKT cells are also able to induce mast cell progenitor accumulation in the lungs through an IL-9 dependent pathway (57). Finally, both prostaglandin E_2_ and leukotriene B_4_ (LTB_4_), which can be highly produced by mast cells, have been shown to enhance chemotaxis of mast cell progenitors (58, 59). In addition to their well-elucidated role in allergic airway disease, there is strong evidence for an accumulation of mast cells in the intestinal tract during helminth infections (60). Furthermore, mast cell precursors appear to accumulate at sites of viral infection including IAV, Sendai virus, infectious bursal disease virus (IBDV), and Newcastle disease virus (NDV) (61–65). Accumulation of mast cell progenitors occurs either in a mast cell degranulation-dependent (24, 62–65) or -independent manner (61, 66). Therefore, mast cell activation can result in the local accumulation of mast cells in infected tissue, further augmenting the role these cells can play during infection (Figure 2).

#### Expression of Pattern-Recognition Receptors by Mast Cells for Sensing Invading Microbes

Mast cells express a large array of innate cell surface and cytosolic receptors that mediate their activation, and as such are integral cells in initiating appropriate immune responses to infectious agents. Notably, mast cells express a large array of Fc receptors including FcϵRI, FcγRI, and FcγRIII (67). Mast cells are also able to respond through a wide variety of pattern-recognition receptors (PRR), including toll-like receptors (TLR), nod-like receptors (NLR), retinoic-acid inducible gene 1-like receptors (RLR), and C-type lectin receptors (CLR), each of which play an essential role in innate immunity by detecting conserved molecular patterns expressed by pathogens (68–82). Mast cells can also be activated through engagement of complement receptors (28), CD48 (83, 84), and integrins (85). Lastly, mast cells can respond to pathogens indirectly through the IL-33 signaling pathway (48). Thus, mast cells are capable of responding to a broad range of pathogen-derived or pathogen-induced stimuli (Figure 1). Interestingly, mast cells do not respond uniformly to all input stimuli (86). For example, signaling through TLR4 leads to a strong pro-inflammatory cytokine response, but limited mast cell degranulation. Conversely, signaling through TLR2 induces both an inflammatory cytokine response and mast cell degranulation (87). Mast cell activation therefore is an important rheostat for the immune system, which will likely modulate to the appropriate response. However, aberrant activation or prolonged activation may elicit tissue immunopathology.

#### Role of Mast Cells in Allergies and Asthma

Mast cells are most frequently recognized for their detrimental role during an allergic response. Following an initial exposure to antigen (Ag), activated B cells can undergo class switching, resulting in the secretion of IgE. The high-affinity IgE receptor, FcϵRI, expressed on the surface of mast cells binds to the Fc portion of IgE, sensitizing the mast cells. Upon subsequent exposures, polyvalent Ag cross-links the surface bound IgE resulting in mast cell degranulation and the production of bioactive lipids and cytokines and chemokines (67, 88, 89).

Mast cells have also been recognized for their role in asthma. Asthma is a pleomorphic disease characterized by recurrent airway restriction, shortness of breath, wheezing, and coughing. Within asthma patients, including both atopic (allergic) and non-atopic (intrinsic), the number, localization, and phenotype of mast cells are altered. Repeated activation of the pulmonary mast cells by the allergen results in mast cells, which are more likely to degranulate compared to non-asthmatic patients (90, 91). Overall, the mast cell response contributes to the bronchial constriction, chronic inflammation, and tissue remodeling typical of asthma patients.

It is now well-documented that infection with respiratory viruses, including IAV, rhinovirus, and respiratory syncytial virus (RSV), often exacerbates asthma (92–96). These upper respiratory tract infections frequently lead to hospital admission for asthma patients (97). Interestingly, asthma was the most common comorbidity among hospitalized patients during the 2009 H1N1pdm IAV pandemic (98–101). A state of hyperresponsiveness in the asthmatic patients, as well as increased levels of inflammatory molecules (e.g., histamine, IL-6, and leukotriene), are believed to contribute to asthmatic exacerbation from viral infection (102). Thus, it is critical we understand the interactions of mast cells with viruses in both naïve hosts and those with chronic inflammatory conditions, which alter mast cell numbers and function.

## Is There a Role for Mast Cell Activation and Mediators during Pathological Viral Infections?

Numerous highly pathological viral infections cause significant disease through immune-mediated pathology to tissue and/or induction of vascular permeability. For example, during dengue virus infections there is significant vascular permeability, which is associated with severe disease and mast cell activity (51, 103). Additionally, severe respiratory virus infection can induce acute respiratory disease syndrome (ARDS), which is associated with significant epithelial–endothelial dysfunction and excessive activation of macrophages and neutrophils (104). ARDS has been observed during experimental IAV infection of animal models, as well as in people naturally infected with highly pathological IAV isolates, such as the 1918 H1N1 “Spanish” influenza strain and the recent zoonotic outbreaks of avian H5N1 and H7N9 IAV strains (105–107). An eloquent transcriptome analysis by Josset et al., which compared highly pathological versus seasonal IAV infections, detected a strong transcriptional signature of macrophages and neutrophils in the lungs of mice with severe IAV infection (108), which fits with prior histological observations (16). Intriguingly, Josset et al. also saw a strong transcriptional contribution of mast cells during these severe IAV infections (108); however, these authors did not explore the role this cell population might play in the observed disease. We propose that, in addition to macrophages and neutrophils, mast cells may contribute to the excessive inflammatory response and vascular problems observed not only during highly pathogenic IAV but also in a range of highly pathogenic viral infections as further discussed below.

### Influenza virus

Pandemic isolates and the emerging highly pathogenic avian strains of IAV are capable of inducing a robust inflammatory response, which causes significant damage within the lungs and the ultimate restructuring of the lung architecture (1). In humans experimentally infected with IAV, detection of histamine metabolites correlates with clinical symptoms (109, 110). Moreover, emerging data in the murine model of IAV suggests a link between mast cell recruitment and activation with lung immunopathology. Following inoculation with a mouse adapted strain of the 2009 H1N1pdm IAV (A/California/04/2009), mice develop significant pathology and inflammation, recapitulating clinical observations from the 2009 pandemic in humans, while mice infected with a non-adapted strain do not (108, 111). In those mice inoculated with the mouse-adapted 2009 H1N1pdm IAV, an enrichment of genes for activated macrophages, neutrophils, and mast cells was observed when compared to mice inoculated with the non-pathogenic strain (108). Moreover, this same observation was made during infection with recombinant 1918 H1N1 (108). Thus, it appears that early accumulation of activated macrophages, neutrophils, and mast cells correlates with the immunopathology associated with pandemic IAV infections.

As this prior transcriptomic study suggested (108), increased mast cell density was observed in the nasal mucosa, trachea, lung parenchyma, and mediastinal lymph node following infection with a highly pathological H5N1 isolate (A/chicken/Henan/1/2004) (24). While these data demonstrated that mast cells are increased in the lungs of mice during highly pathological IAV infection, their role in the inflammatory response induced by IAV remained elusive. In this regard, recent data demonstrates that mast cells can play a detrimental role during IAV infection in a strain specific manner. Specifically, following infection with A/WSN/1933, B6.Cg-*Kit^*W-sh*^* mice, which lack mast cells (112), exhibit a reduction in weight loss, lung pathology, and pulmonary inflammation compared to wild-type mice (23). Importantly, when mast cells are reconstituted into B6.Cg-*Kit^*W-sh*^* mice, the weight loss and inflammatory response are restored to wild-type levels (23). In studies using a highly pathogenic H5N1 virus (A/chicken/Henan/1/2004), mice administered ketotifen, a mast cell stabilizing agent, demonstrate reduced lung inflammation and epithelial cell apoptosis than untreated mice (24). Furthermore, combination therapy with ketotifen and oseltamivir (an NA inhibitor) improves survival better than either drug alone (24). Taken together, these data show mast cells can contribute to the pathology observed during IAV infection in mice. The newly emerging zoonotic strains of highly pathogenic IAV, such as H7N2, are also presenting with high cellular infiltrate and damage within the lungs of mice, suggestive of mast cell activation (25, 107). If mast cells participate in the immunopathology elicited by these emerging zoonotic IAV isolates remains to be seen.

### Dengue virus

Human infection with dengue virus can result in a wide range of pathologies. In its most severe forms, dengue virus induces dengue hemorrhagic fever and dengue shock syndrome, both of which are characterized by increased vascular permeability. The production of cross-reactive antibodies during a primary infection can lead to more severe disease upon secondary infection with a heterologous serotype (113, 114). The urine and blood of infected patients display elevated levels of histamine (115, 116), the presence of vasoactive factors (117, 118) and increased serum levels of chymase, a mast cell specific enzyme (103). As each of these mediators is released by mast cells, numerous studies have examined the role mast cells play during dengue virus infection. Upon exposure, dengue virus induces both degranulation and cytokine production by mast cells (82, 103, 119, 120). Mast cell derived LTB_4_ and granule proteases increase vascular permeability (82, 103), while the synthesis and release of TNF-α, IL-6, IFN-α, CCL2, CCL3, CCL5, and CX3CL1 recruit NK cells and T cells to the site of infection (82, 121–123). Mast cell deficient mice show a reduction in symptoms, demonstrating that mast cells play an important role in dengue virus-induced immunopathology (103). Moreover, administration of the mast cell stabilizing drugs, cromolyn and ketotifen, or the LTB_4_ antagonist montelukast results in reduced vascular leakage compared to untreated mice (103). Current data suggests that early after infection, mast cell activation by dengue virus is beneficial, as it recruits NK and T cells to promote viral clearance (82, 122, 123). However, widespread mast cell activation is detrimental, as it increases vascular leakage, leading to the more severe forms of dengue-induced disease (103). In a murine model, the presence of non-neutralizing IgG enhances mast cell degranulation during dengue infections through interactions with FcγRIII (124). Therefore, dengue virus can activate mast cells both directly, through an as yet unidentified mechanism, or indirectly through FcγRIII.

### Hantavirus

The zoonotic transmission of hantavirus to humans can result in hemorrhagic fever with renal syndrome or hantavirus cardiopulmonary syndrome, both of which are characterized by increased vascular permeability and thrombocytopenia (125). Patients with hemorrhagic fever with renal syndrome exhibit significantly elevated histamine levels, indicating a possible role for mast cells in potentiating this syndrome (125). Endothelial cells, epithelial cells, and dendritic cells are all permissive to hantavirus infection *in vitro* (125–127), and recent evidence suggests mast cells are also susceptible to this virus (125). Inoculation of *in vivo* differentiated mast cells results in productive infection and mast cell activation, though the ability of hantavirus to directly induce degranulation is not known (125). Furthermore, the ability of various strains of hantavirus to infect and replicate within mast cells directly correlates with the pathogenicity of the strains (125). Thus, mast cells may be an important factor during hantavirus-induced disease.

### Sendai virus

Sendai virus is a respiratory parainfluenza virus that is highly transmissible in both rodents and swine. In neonatal rats, Sendai virus causes viral bronchiolitis and airway hyperresponsiveness, which are associated with elevated levels of bronchiolar mast cells and eosinophils (66, 128–130). The elevated numbers of bronchiolar mast cells observed after Sendai virus infection result from both the proliferation of tissue-resident mast cells and recruitment of mast cell progenitors to the airways (61). Sendai virus can also infect human mast cells, resulting in their activation (131). While the release of β-hexosaminidase (a major granule component) has not been detected from human mast cells, both histamine release in rats and tryptase release in pigs have been detected following Sendai virus challenge (131–133). Following Sendai virus infection, human mast cells produce type I and III interferon (131), which have been implicated in asthma exacerbations (134). Interestingly, in the rat model, animals previously infected with Sendai virus subsequently sensitized to ovalbumin 1-month later display heightened allergic airway inflammatory cell reactions (66). Thus, mast cells are important contributors to the inflammatory response to parainfluenza viruses, and participate in their pathological role during allergic airway disease.

### Infectious bursal disease virus (IBDV)

IBDV is a contagious disease with a high mortality rate, which impacts the poultry industry worldwide. IBDV infected chickens have increased inflammatory lesions, which lead to susceptibility to secondary infections (135–137). Mast cell numbers are increased at the site of infection during IBDV. Moreover, these mast cells are activated, as mast cell tryptase accumulates in the infected tissue (64). Treatment with ketotifen not only decreases mast cell numbers in infected birds but also correlates with reduced injury during infection without altering expression of IBDV Ags (65). Thus, by reducing the release of mast cell mediators, one can decrease mast cell accumulation in the infected tissue, and ultimately decrease tissue damage, and increase survival during IBDV infection.

### Newcastle disease virus (NDV)

NDV is another highly contagious poultry disease, which infects the gastrointestinal tract, resulting in high mortality and economic losses (138). Similar to IBDV, mast cells are found in and around NDV lesions during infection, correlating with an increase of mast cell tryptase levels in the tissues (63). Chickens pretreated with ketotifen show a reduction in tissue damage during NDV infection (62). Thus, similar to IBDV, inhibition of mast cell mediators reduces mast cell accumulation in the infected tissue and decreases tissue damage, increasing survival following NDV infection.

### Porcine reproductive and respiratory syndrome virus

Porcine reproductive and respiratory syndrome virus (PRRSV) is associated with high mortality in pigs. Infection with low pathogenic PRRSV (LP-PRRSV) results in minimal histopathological changes with no mortality. In contrast, infection with a high pathogenic strain of PRRSV (HP-PRRSV) results in significant mortality associated with extensive tissue damage within the lungs (139, 140). Pigs infected with HP-PRRSV display significant respiratory distress, which is associated with pulmonary lesions characterized by inflammatory cell infiltrates, interstitial and alveolar edema, and hemorrhaging, which is not observed following LP-PPRSV infection. Infection with the HP-PRRSV results in higher virus titers and higher levels of pro-inflammatory cytokines and immune cell infiltrate, including neutrophils, mononuclear phagocytes, and mast cells. Both histamine and LTB_4_ are significantly increased in the serum of HP-PRRSV infected pigs (141). Because, these mediators play an important role increasing blood vessel permeability and disease severity during dengue virus infection (51), it is likely they contribute to the increased lung edema and hemorrhage observed during HP-PRRSV (141).

## How are Mast Cells Activated by Viruses?

### Are virus entry and replication in mast cell required for activation?

Both pathogenic and non-pathogenic hantavirus nucleoprotein can be detected in mast cells (125). In addition, the human mast cell lines KU812 and HMC-1 are permissive to dengue virus in the presence of human dengue virus immune sera (119). This data demonstrate that these highly pathogenic viruses can infect mast cells. RSV activates mast cells resulting in the production of cytokines and chemokines including CXCL10, CCL4, CCL5, and type I interferons (142). RSV Ag can be detected in both primary cord blood mast cells and the human mast cell lines following infection (142). However, similar to many other pathogenic viruses, mast cell infection does not result in the release of infectious progeny virions (142).

While respiratory epithelial cells are the primary target for IAV replication, IAV can infect a wide range of cells, including endothelial cells (21), macrophages (143), dendritic cells (144), and mast cells (23, 121, 145). In mast cells, IAV is able to mediate viral entry, but largely appears to undergo an abortive infection. Inoculation of murine bone marrow derived mast cells (BMDMC) with A/WSN/1933 results in *de novo* expression of the viral NS-1 protein, but does not produce any new infectious particles (23). Interestingly, treatment of murine BMDMC with another H1N1 isolate, A/PR/8/1934, does not result in detectable NS-1 expression (23). On the other hand, infection of the human mast cell line LAD and human cord blood derived mast cells with the A/PR/8/1934 strain results in viral mRNA and protein synthesis, but does not produce *de novo* infectious particles (145). In contrast, recent data demonstrate the murine mastocytoma cell line P815 can be productively infected with A/WSN/1933 (H1N1), A/Chicken/Henan/1/2004 (H5N1), and A/Chicken/Hebai/2/2002 (H7N2), producing infectious virus over the first 24 h of infection, as measured by qRT-PCR, hemagglutination assay, and plaque forming assay (25). These differences likely reflect the different types of mast cells used for these studies and the infectious dose of the virus. Overall, these data demonstrate that IAV, dengue virus, RSV, and hantavirus can at least bind to and enter mast cells, which is likely important for mast cell activation. More studies are needed to understand the fine specificity of these viruses, and specifically the different IAV isolates for distinct mast cell populations, and the cellular factors that may be present in some of these populations that limit IAV propagation.

### How are viral particles recognized by mast cells?

Mast cells express a wide range of PRR which allows these cells to respond to a variety of stimuli, including bacteria, parasites, fungi, and viruses (Figure 1) (86). RIG-I is a cytosolic receptor that can detect IAV RNA and many other single stranded RNA viruses (73, 80). Once RIG-I detects vRNA, it signals through the mitochondrial adaptor MAVS resulting in an antiviral response. In mast cells, signaling through the RIG-I/MAVS pathway is important for the secondary response to IAV, but not for the immediate degranulation of mast cells (23). Virus recognition through RIG-I by mast cells is also important during dengue virus and vesicular stomatitis virus (VSV) infections (82, 121, 146). However, our studies suggest the RIG-I dependent responses in mast cells do not significantly contribute to the pulmonary immunopathology associated with IAV infection (Graham and Obar, unpublished observation); rather, mast cell degranulation appears to be the dominant mediator of immunopathology (24). In addition to RIG-I detection, TLR3 is also important for the recognition of IAV, type I reovirus, RSV, VSV, and NDV by murine BMDMC for the production of secondary mast cell mediators (79, 147, 148). Moreover, viral recognition by both Mda5 and 2′-5 oligoadenylate synthase (OAS) can participate in the initiation of the secondary response of mast cells induced by VSV (148). Thus, detection of viral nucleic acids appears to be central for production of *de novo* synthesized mast cell mediators following viral infection. Alternatively, infection can be detected indirectly by mast cells, as occurs with herpes simplex virus (HSV). Infected epithelial cells secrete IL-33, which is in turn detected by mast cells, resulting in the secretion of IL-6 and TNF-α without degranulation (149).

Mast cell degranulation not only appears to play a critical role in regulating mast cell dependent inflammation following IAV infection (23, 24) but also in a number of other viral systems (62, 65, 103). The mast cell degranulation inhibitor, ketotifen, reduces inflammation in response to H5N1 IAV infection of mice (24), and the inflammation associated with IBDV and NDV in poultry (62, 65). Additionally, mast cell stabilization using cromolyn limits dengue virus induced immunopathology (103). Together, these data strongly support a role for mast cell degranulation in the mast cell-dependent inflammatory response to highly virulent viral infections. Thus, it appears critical we understand how viruses drive mast cell degranulation to appropriately target these cells pharmacology.

How mast cells degranulate in response to viral infections remains largely unknown. Degranulation still occurs in response to A/WSN/1933 infection in RIG-I-deficient BMDMC, demonstrating that degranulation is a RIG-I-independent response (23). As degranulation occurs within 30 min following treatment with IAV, other PRR and/or early signaling events necessary for the virus attachment and/or entry processes are likely important in regulating mast cell degranulation. With dengue virus, degranualtion of mast cells occurs prior to RIG-I signaling (82). Moreover, UV-inactivated dengue virus (82) and IAV (147) retain the ability to activate mast cells, suggesting this occurs early in the viral replication cycle. While FcγIII-deficient mast cells are able to degranulate in response to dengue virus, mast cells pre-treated with anti-dengue IgG demonstrate enhanced degranulation in response to all four serotypes of dengue virus compared to dengue virus alone, suggesting that antibody binding enhances degranulation in response to dengue virus (124). Although mast cell degranulation appears to be pivotal for the immunopathology associated with highly pathological IAV (24) and dengue virus infections (103), we do not understand how degranulation is initiated. To date, the only virus for which the mechanism of mast cell degranulation has been well elucidated is vaccinia virus. The activating event is fusion of the viral envelope with the mast cell plasma membrane (31). Specifically, the vaccinia virus envelope contains sphingomyelin (150), which is converted to sphingosin-1-phosphate (S1P) and signals through the S1PR2 G-coupled receptor to cause degranulation (31). Signaling through the S1PR2 has also been shown to regulate mast cell responses in general (31, 151–154). However, the role of S1P receptor signaling in other viral infections remains unknown. Further understanding the molecular signals necessary for mast cell degranulation could lead to novel therapeutic avenues for these highly virulent viral infections.

## Mast Cells as Drug Targets for Limiting Virus-Induced Immunopathology

Predicting the next pandemic IAV strain is nearly impossible, as IAV has a high mutation rate resulting in significant yearly antigenic drift and can randomly reassort resulting in antigenic shift. Even deciding which IAV strains to produce for the yearly vaccine is difficult, as the strains must be chosen months ahead of the yearly influenza season. If these predictions are inaccurate or the seasonal IAV strains drift significantly, then the vaccine will not be highly effective resulting in a high incidence of IAV-induced disease (2). The current antiviral treatments against IAV are becoming increasingly ineffective due to the emergence of resistant strains. Therefore, alternative therapeutics avenues are needed. Targeting host-derived factors necessary for viral replication or host factors participating in the excessive pathological inflammatory response during highly pathogenic IAV are promising alternatives (2).

**Figure 3:**
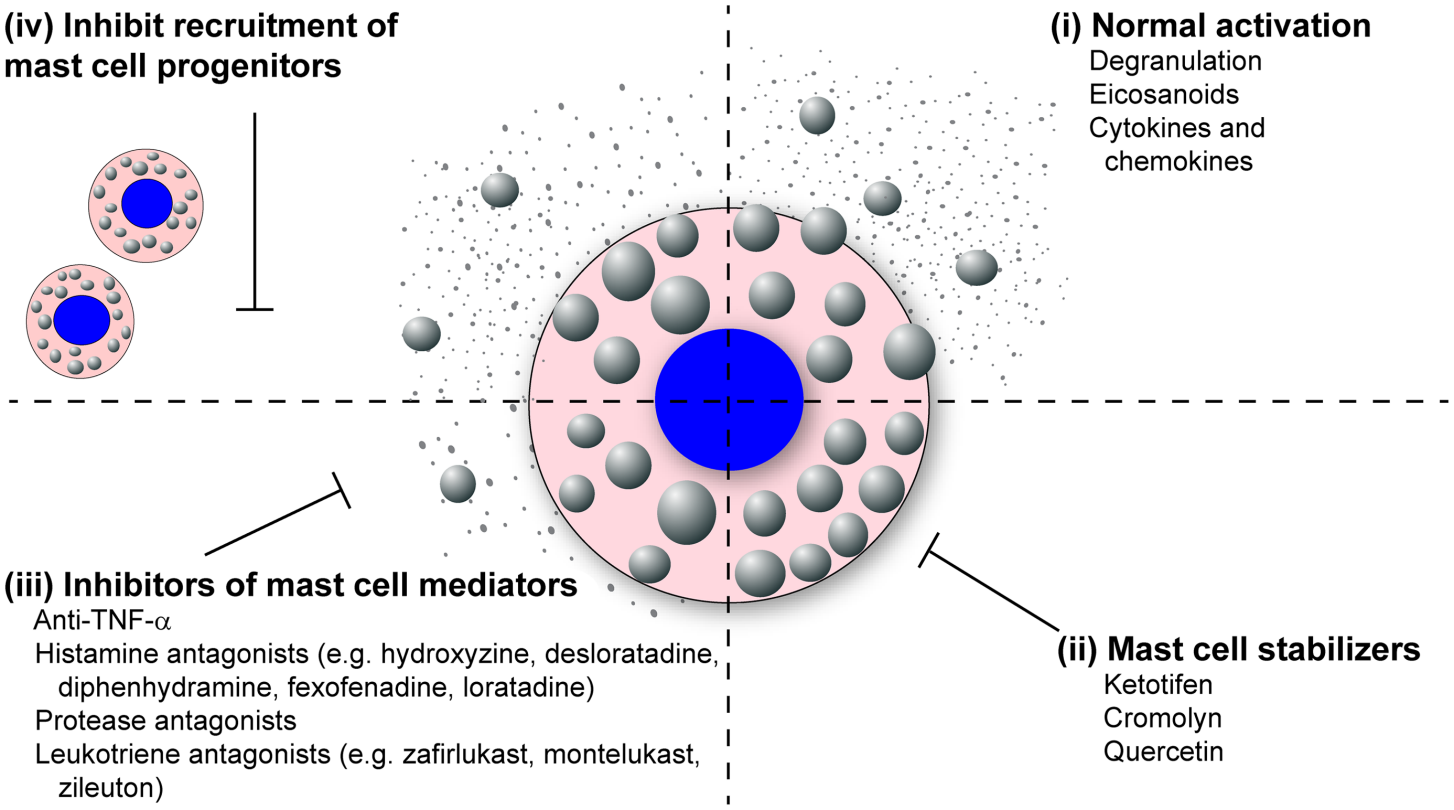
**Mast cell inhibitors**. Various classes of mast cell inhibitors already exist for the treatment of various conditions. (i) Uninhibited, activated mast cells will degranulate and synthesize eicosanoids, cytokines, and chemokines which are released into the surrounding tissue. (ii) The mast cell stabilizing drugs (e.g., ketotifen, cromolyn, and quercetin) block the release of mast cell granules following activation. (iii) Second broad class of mast cell inhibitors target the activity of specific mast cell mediators. These includes anti-TNF-α compounds, anti-histamines (e.g., hydroxyzine, desloratadine, diphenhydramine, fexofenadine, loratadine), protease antagonists, and leukotriene antagonists (e.g., montelukast, zafirlukast, zileuton). (iv) A potential third class of mast cell inhibitors could target the recruitment of mast cells to inflamed tissue following infection.

The literature review presented here shows the strong correlation between mast cell accumulation and degranulation at local sites of infection with the observed tissue damage and pathology, not only during highly pathological IAV infections but many other pathogenic viral infections of humans and animals. Additional studies examining other highly pathological viruses that are known to cause ARDS and/or vascular leakage are thus warranted, which would include the emerging coronaviruses, SARS-CoV, and MERS-CoV, and hemorrhagic viruses such as Marburg and Ebola. Overall, we hypothesize that excessive mast cell activation may be a common feature of highly pathological viral infections that cause ARDS and/or vascular leakage. This novel pathway could be pharmacologically targeted to limit the morbidity and mortality associated with these infections. Additionally, understanding how mast cells accumulate in the infected tissues, through mast cell proliferation and/or mast cell progenitor recruitment, could provide additional therapeutic targets (Figure 3).

Because mast cells and their products are known to play a dominant role in both allergic and asthmatic reactions, many drugs that stabilize and neutralize mast cells are already approved for human use (Figure 3). The mast cell stabilizing drugs, which inhibit the release of granules following mast cell activation, have proven effective at reducing vascular leakage and limit inflammatory cellular recruitment, thus increasing survival in the murine dengue virus and IAV models (24, 103, 155). Furthermore, these compounds have proven very effective at limiting lung pathology following IBDV and NDV in poultry (62, 65). Compounds are also available which block the activity of specific mast cell products including TNF-α, histamine, mast cell proteases, and leukotrienes (Figure 3). Many anti-TNF-α compounds are already approved for the treatment of inflammatory arthritis. Numerous anti-histamines, including hydroxyzine, desloratadine, diphenhydramine, fexofenadine, and loratadine, are approved to treat allergy symptoms. Drugs are currently in development, which target the mast cell proteases, especially the mast cell derived chymase which has been implicated in cardiovascular disease. Finally, there are two classes of leukotriene antagonists, the leukotriene-receptor antagonists (zafirlukast and montelukast) and the leukotriene synthesis inhibitors (zileuton).

In addition to stand alone treatments targeting mast cell activation and mediators, adjunct therapies utilizing both antiviral and mast cell targeting compounds might be fruitful. Earlier studies using human peripheral blood leukocytes exposed to NAs or IAV at the time of IgE stimulation resulted in significantly greater histamine release (156–158). These data suggest the presence of multiple stimuli may result in an additive or synergistic effect. Therefore, mast cell targeting drugs could be used in parallel with antiviral drugs for greatest efficacy. Following infection with a highly pathogenic H5N1 IAV strain, the only cohort of mice which survived infection were those treated with both antiviral and mast cell stabilizing compounds (24). This approach may prove especially beneficial during asthmatic exacerbations following viral infection.

## Concluding Remarks

Mast cells are important players in pathogen defense. Their location at environmental barriers allows them to quickly respond to invading pathogens. In parasitic and bacterial infections, mast cells are essential in preventing the spread of infection (26–28). While in certain viral infections mast cells can be protective (31, 122, 123, 149), in highly pathogenic viral infections, such as IAV or systemic dengue infections the data demonstrate that mast cells are more detrimental than beneficial (23, 24, 103). If the role of mast cells during IAV infections, and other highly pathogenic viral infections, can be elucidated, these cells may serve as a lucrative target for new therapeutics. Activation and release of mediators from mast cells in response to these viruses correlates with severity of disease in mice. Application of existing allergy medications that target mast cells may decrease the severity of IAV infections, limiting the morbidity and mortality associated with future pandemics.

## Conflict of Interest Statement

The authors declare that the research was conducted in the absence of any commercial or financial relationships that could be construed as a potential conflict of interest.
